# Bayes Lines Tool (BLT): a SQL-script for analyzing diagnostic test results with an application to SARS-CoV-2-testing

**DOI:** 10.12688/f1000research.51061.2

**Published:** 2021-06-18

**Authors:** Wouter Aukema, Bobby Rajesh Malhotra, Simon Goddek, Ulrike Kämmerer, Peter Borger, Kevin McKernan, Rainer Johannes Klement

**Affiliations:** 1 Independent Data and Pattern Scientist, Hoenderloo, 7351BD, The Netherlands; 2 Department for Digital Arts, University for Applied Arts Vienna, Vienna, 1030, Austria; 3 Independent Scientist, Ede, 6711 VS, The Netherlands; 4 Department of Obstetrics and Gynaecology, University Hospital of Würzburg, Würzburg, 97080, Germany; 5 The Independent Research Initiative on Information & Origins, Loerrach, 79540, Germany; 6 Medical Genomics, Beverly, MA, 01915, USA; 7 Department of Radiation Oncology, Leopoldina Hospital Schweinfurt, Schweinfurt, 97422, Germany

**Keywords:** Bayes, COVID19, PCR Test, SARS-CoV-2; SQL

## Abstract

The performance of diagnostic tests crucially depends on the disease prevalence, test sensitivity, and test specificity. However, these quantities are often not well known when tests are performed outside defined routine lab procedures which make the rating of the test results somewhat problematic. A current example is the mass testing taking place within the context of the world-wide SARS-CoV-2 crisis. Here, for the first time in history, laboratory test results have a dramatic impact on political decisions. Therefore, transparent, comprehensible, and reliable data is mandatory. It is in the nature of wet lab tests that their quality and outcome are influenced by multiple factors reducing their performance by handling procedures, underlying test protocols, and analytical reagents. These limitations in sensitivity and specificity have to be taken into account when calculating the real test results. As a resolution method, we have developed a Bayesian calculator, the Bayes Lines Tool (BLT), for analyzing disease prevalence, test sensitivity, test specificity, and, therefore, true positive, false positive, true negative, and false negative numbers from official test outcome reports. The calculator performs a simple SQL (Structured Query Language) query and can easily be implemented on any system supporting SQL. We provide an example of influenza test results from California, USA, as well as two examples of SARS-CoV-2 test results from official government reports from The Netherlands and Germany-Bavaria, to illustrate the possible parameter space of prevalence, sensitivity, and specificity consistent with the observed data. Finally, we discuss this tool’s multiple applications, including its putative importance for informing policy decisions.

## 1. Introduction

In December 2019, a cluster of patients with pneumonia of unknown origin was associated with the emergence of a novel beta-coronavirus of bat origin, ^1^ first named 2019-nCoV ^2^ and later specified as severe acute respiratory syndrome-coronavirus-2 (SARS-CoV-2). ^3^ This outbreak led to the rapid development of reverse transcriptase - quantitative polymerase chain reaction (RT-qPCR) tests to identify SARS-CoV-2 RNA in specimens obtained from patients. ^2, 4^

After sporadic SARS-CoV-2 positive cases in January ^5, 6^ to the end of February 2020 worldwide cases of the SARS-CoV-2-associated disease ‘COVID-19’ began to accumulate, causing policymakers in many countries to introduce countermeasures. These Non-Pharmaceutical Interventions (NPIs) predominantly started worldwide around March 2020 while the virus was characterized as a pandemic on 11 March, 2020. ^6, 7^ As a result, for more than one year now, large parts of the world are in a COVID-19 crisis-mode with daily reporting of SARS-CoV-2 cases in dashboards worldwide. ^8^ The definition of ‘cases’ and ‘prevalence estimates’ was based on RT-qPCR testing, independent of the clinical diagnosis. Thereby, a person is considered a case (i.e., infected), once a test turns out positive. ^9^

Like all laboratory tests, however, the SARS-CoV-2 RT-qPCR tests are not flawless. This is because sensitivity and specificity depend on a multiplicity of confounding factors. These factors cover the test design, the lab application, and possible contaminations with substances/nucleic acids interfering with the reaction. ^10, 11^ Consequently, both false-negative and false-positive results have been reported. ^12, 13^ Nevertheless, the test system’s limitations are rarely discussed in scientific publications and public health systems despite their crucial role for making inferences about the possible infection status of a tested person. ^14^ Many more or less defined commercial and laboratory ‘in house’ tests are now routinely being used, ^15^ often without standardised guidelines, which leads to entirely unknown test performance specifications. ^16^ The few studies aiming to estimate sensitivity and specificity of SARS-CoV-2 RT-qPCR tests have reported sensitivities and specificities in the ranges ≳30% and ≳80%, respectively - therefore, the communicated data seldom can offer precise distinctions. ^14^

Given the critical role that dashboards and graphs based on SARS-CoV-2 test results play for policymakers, health professionals, and the general public, ^8^ our objective was to develop a Bayesian calculator that could calculate test quantities and prevalence solely based on officially reported numbers of total and positive tests, i.e., without making any *a priori* assumptions. In this way, time trend estimates and country-to-country comparisons of these test performance measures as well as disease prevalence estimates become possible, producing in-depth insights, making projections/simulations possible, and providing a more holistic understanding of the daily incoming data in general.

## 2. Methods

### 2.1 General description of the calculator

The Bayes Lines Tool (BLT) calculator is based on Bayes’ theorem and estimates the true and false positive, and true and false negative numbers at a given time point for which the total number of tests performed and the number of positive test results is known. These data are usually reported and published by official government bodies daily and/or weekly. Thus, the model uses the following information:

• Publishing date or report identifier of the test data
• Number of performed tests (#tests)
• Number of reported positive results (#positives)

The model takes this information as a given fact and uses it to make inferences about the test performance parameters (sensitivity and specificity) as well as the prevalence (also known as the base rate) - these inferences are essential for estimating the number of true positives (TP), false positives (FP), true negatives (TN) and false negatives (FN). It is assumed that there is no knowledge of either the prevalence or the sensitivity and specificity of the tests used. Instead, the model explores all possible combinations of two of these three parameters within reasonable ranges specified by the user; for each of these combinations, the third parameter can then be calculated using the dependencies through Bayes’ theorem. Finally, all parameter combinations that result in TP+FP estimates consistent with the known number of positive tests are selected and stored as Bayesian confusion matrices (CMs).

A single CM contains TP, FP, TN, and FN in absolute numbers ( Table 1). For a given prevalence, sensitivity, and specificity these are derived from Bayes’ theorem:

(1) $$P\left(I|T\right)=\frac{P\left(T|I\right)\times P\left(I\right)}{P\left(T\right)}$$

**Table 1.** A confusion matrix for a SARS-CoV-2 test containing absolute numbers of true (TP) and false (FP) positives and true (TN) and false (FN) negatives derived from equations ( 2)-( 5).

Actual infection status	Test result positive	Test result negative
INFECTED	TP	FN
NOT INFECTED	FP	TN

Here, T denotes the hypothesis that a test comes out positive ( ¬T its denial) and I the hypothesis that an individual is infected, so that $P\left(I\right)$ is the prevalence and $P\left(T|I\right)$ is the test sensitivity. $P\left(T\right)$ is the marginal probability of a positive test, which we estimate as the frequency of positive test results, whereas $P\left(I|T\right)$ is the probability of being infected given that the test came out positive. With the normalizing constant $P\left(T\right)$ estimated as $P\left(T\right)=\frac{\#\text{positives}}{\#\text{tests}}$ and $P\left(I|T\right)$ estimated as the proportion of infected individuals among those in which the test came out positive, equation (1) becomes:

(2) $$\mathrm{TP}=P\left(I|T\right)\times \#\text{positives}=\text{sensitivity}\times \text{prevalence}\times \#\text{tests}$$

Equation (2) thus shows that the number of TPs depends on the prevalence, test sensitivity and total number of tests performed. Using $P\left(\neg T|\neg I\right)$ =specificity and #negatives = #tests−#positives, an analogous derivation leads to

(3) $$\mathrm{TN}=P\left(\neg I|\neg T\right)\times \#\text{negatives}=\text{specificity}\times \left(1-\text{prevalence}\right)\times \#\text{tests}$$

From Equations (2) and (3), FP and FN follow as

(4) FP=#positives−TP

(5) FN=#tests−#positives−TN

### 2.2 Implementation

For the implementation presented here, the two parameters which varied are as follows:

• Sensitivity from 0.005 to 1 with 0.005 increments.
• Specificity from 0.005 to 1 with 0.005 increments.

For a given sensitivity and specificity as well as number of tests and positives, the prevalence can then be computed as

(6) $$\text{prevalence}=\frac{\left(\frac{\#\text{positives}}{\#\text{tests}}+\text{specificity}-1\right)}{\text{sensitivity}+\text{specificty}-1}$$

Hereby, calculations for combinations of sensitivity and specificity that add to ≤1 are omitted, and cases in which prevalence turns out negative or larger than 1 are discounted as unphysical.

We developed an SQL query that generates all possible Bayesian CMs for a series of diagnostic test results, without making assumptions about prevalence, sensitivity, or specificity.

The code in PostgreSQL is given as follows (Code 1):


with tests as (
    select
   :reg :: text as region_name,
   :rid :: text as report_id,
   :tst :: float as tests,
   :pos :: float as positives
 ),

permutations as (
    select
   sens :: float as sensitivity,
   spec :: float as specificity
 from
   generate_series(0.005, 1.000, 0.005) as sens,
   generate_series(0.005, 1.000, 0.005) as spec
 ),

prevalences as (
    select
   (positives/tests + specificity - 1) :: float/
         (sensitivity + specificity - 1) :: float as prevalence,
   *
 from
  permutations,
  tests
 where
  sensitivity + specificity > 1

 ),

matrices as (
 select
  (tests * prevalence * sensitivity) :: float as true_positives,
  (tests * (1 - prevalence) * specificity) :: float as true_negatives,
  *
 from
  prevalences
 where
  prevalence between 0 and 1
 ),

results as (
 select
  positives - true_positives as false_positives,
  (tests - positives) - true_negatives as false_negatives,
  *
 from
  matrices
 )

select
 region_name,
 report_id,
 (tests) :: int             as tests_performed,
 (positives) :: int           as positives_reported,
 (tests * prevalence) :: int     as has_disease,
 (tests * (1 - prevalence)) :: int  as hasnot_disease,
 (true_positives) :: int        as true_positives,
 (false_positives) :: int       as false_positives,
 (true_negatives) :: int        as true_negatives,
 (false_negatives) :: int       as false_negatives,
 sensitivity :: numeric(4,3),
 specificity :: numeric(4,3),
 prevalence :: numeric(4,3)
from
 results
where
 (false_positives + true_positives) :: int = positives :: int



Given the test results published in the databases and given all generated permutations and consequently all possible CMs, only those are returned that match the positive test results. With only the resulting CMs for which TP+FP match the positives reported in the input data, we are able to identify patterns that provide additional insights for further investigation.

In order to produce CMs for a series of reports, such as daily test result numbers, several approaches are possible. In this manuscript we describe a practical application for using a Batch/Script approach. The Script is used on Apple OSX, the example below using COVID-19 data from the Netherlands (Code 2):


psql -h localhost -d postgres -U postgres -A --set=rid=\'20200601\' --set=reg=\'Netherlands_GGD\' --set=tst=1552--set=pos=73 -f BLTV3.sql >> Netherlands_GGD.txt
psql -t -h localhost -d postgres -U postgres -A --set=rid=\'20200602\' --set=reg=\'Netherlands_GGD\' --set=tst=6819 --set=pos=203 -f BLTV3.sql >> Netherlands_GGD.txt
psql -t -h localhost -d postgres -U postgres -A --set=rid=\'20200603\' --set=reg=\'Netherlands_GGD\' --set=tst=8867 --set=pos=165 -f BLTV3.sql &gt;&gt; Netherlands_GGD.txt
psql -t -h localhost -d postgres -U postgres -A --set=rid=\'20200604\' --set=reg=\'Netherlands_GGD\' --set=tst=9339 --set=pos=171 -f BLTV3.sql &gt;&gt; Netherlands_GGD.txt
psql -t -h localhost -d postgres -U postgres -A --set=rid=\'20200605\' --set=reg=\'Netherlands_GGD\' --set=tst=9464 --set=pos=135 -f BLTV3.sql &gt;&gt; Netherlands_GGD.txt
psql -t -h localhost -d postgres -U postgres -A --set=rid=\'20200606\' --set=reg=\'Netherlands_GGD\' --set=tst=7843 --set=pos=125 -f BLTV3.sql >> Netherlands_GGD.txt
psql -t -h localhost -d postgres -U postgres -A --set=rid=\'20210224\' --set=reg=\'Netherlands_GGD\' --set=tst=52551 --set=pos=4374 -f BLTV3.sql >> Netherlands_GGD.txt



### 2.3 Data

For the examples demonstrated in the Results section below, we extracted test data from:

- A hypothetical scenario used for assessing the performance of BLT and demonstrating the so-called spectrum effect ^17, 18^
- Influenza data for the Californian Bay Area obtained from the California Open Data Portal at
https://data.ca.gov/dataset/influenza-surveillance/resource/d2207905-14eb-4264-9a02-8b6ac15ddc39?inner_span=True
- The Netherlands/Dutch Corona Dashboard database, used as examples for a daily report and a time trend analysis:
https://coronadashboard.rijksoverheid.nl/landelijk/positief-geteste-mensen
- The German LGL Bayern database, derived from RKI (Robert Koch Institute) data: https://www.lgl.bayern.de/gesundheit/infektionsschutz/infektionskrankheiten_a_z/coronavirus/karte_coronavirus/

## 3. Results

In the following section examples are provided that demonstrate the application of our calculator for the data referenced in Section 2.3.

### 3.1 A hypothetical scenario

Consider the following hypothetical scenarios displayed in Table 2 that we used for a general check of BLT’s performance. In scenarios 1 and 2, we consider a disease which has a prevalence of 20% in two different subpopulations (e.g. young and old people, respectively). Each subpopulation has its own test characteristics: In subpopulation 1, test sensitivity is 95% and specificity 75%, while in subpopulation 2, sensitivity is 75% and specificity 95%. Consider that 10,000 tests have been performed in the total population. In scenario 1, the total population consists of an equal mix of both subpopulations, while in scenario 2 the total population consists of 75% subpopulation 1. The different mixture of subpopulations leads to a different number of positive test results, and hence a different input for BLT. The overall test performance measures (sensitivity and specificity) are a weighted average between the subpopulation test performance measures. This is called the spectrum effect. ^17^

**Table 2.** A hypothetical testing scenario.

Estimation	TP	TN	FP	FN	Prevalence [%]	Sensitivity [%]	Specificity [%]
Scenario 1: Balanced distribution of population 1 and 2							
Overall	1700	6800	1200	300	20	85	85
Population 1	950	3000	1000	50	20	95	75
Population 2	750	3800	200	250	20	75	95
Scenario 2: Unbalanced distribution of populations: 75% population 1							
Overall	1800	6400	1600	200	20	90	80
Population 1	1425	4500	1500	75	20	95	75
Population 2	375	1900	100	125	20	75	95
Scenario 3: Balanced distribution of populations with different prevalence							
Overall	2410	7075	490	25	24.35	99.0	93.5
Susceptible	1900	2880	200	20	38.4	99.0	93.5
Less susceptible	510	4195	290	5	10.3	99.0	93.5
Scenario 4: Unbalanced distribution of populations with different prevalence							
Overall	2117	7046	783	54	21.7	97.5	90.0
Susceptible	780	1080	120	20	40.0	97.5	90.0
Less susceptible	1337	5966	663	34	17.1	97.5	90.0

Total number of tests is 10,000 in all scenarios. Note that in scenarios 1, 3 and 4, the total number of positives is 2900. TP: True positive number; TN: True negative number; FP: False positive number; FN: False negative number.

Now consider a different scenario, in which the total population is a mix between two subpopulations with different susceptibility towards the disease, and hence different prevalence, but the test performs equally well in both subpopulations. In scenario 3, each subpopulation contributes 50% to the overall population, while in scenario 4, the less susceptible population contributes 80% (8,000 tests). Now the overall prevalence is the weighted average of the subpopulation prevalence values, and overall test sensitivity and specificity are equal to those of the subpopulations.

**Figure 1.**
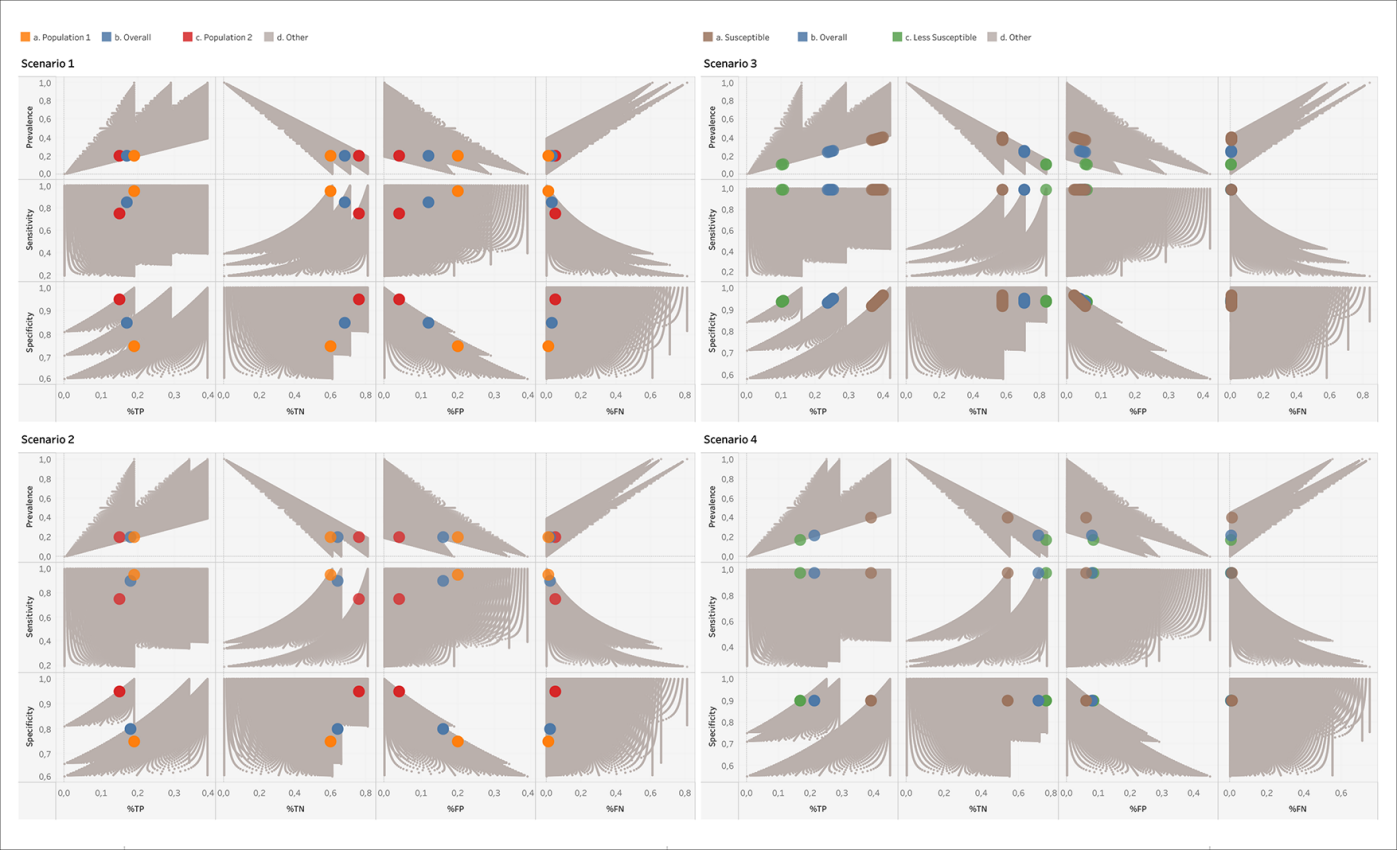
Results of running BLT on the four scenarios given in Table 2. The correct solutions corresponding to the overall and subpopulations of these scenarios are highlighted as large colored points, while all other solutions compatible with the number of tests and positives are shown in grey. For scenario 3, no exact match of prevalence, sensitivity and specificity to the TP and FP numbers could be obtained with the step sizes used in Code 1, so that we display the closest matches. %TP, %TN, %FP, %FN: Percentages of TP, TN, FP and FN numbers relative to the total number of tests performed.

Figure 1 displays all solutions that BLT delivers for scenarios 1-4, with the known solutions of the overall and subpopulations highlighted. It is visible that the spectrum effect observed in Table 2 is also visible in Figure 1, as it translates into the percentages of TPs, TNs, FPs and FNs. What is critical is the fact that BLT, which only works with the total number of tests and positives obtained, would not be able to distinguish between scenarios 1, 3 and 4. All three are compatible with the output set of CMs. One should thus keep in mind for the interpretation of BLT’s output that the solution corresponding to reality is determined by the mix of subpopulations being tested, which in turn might have their own specific subpopulation prevalence, sensitivity and specificity values. In other words, one should be aware of the spectrum effect. ^17, 18^ If possible, one should thus use knowledge about prevalence and test performance measures to filter out the CMs consistent with what is known about “the reality”.

### 3.2 California/USA (diagnostic Influenza-testing)

**Figure 2.**
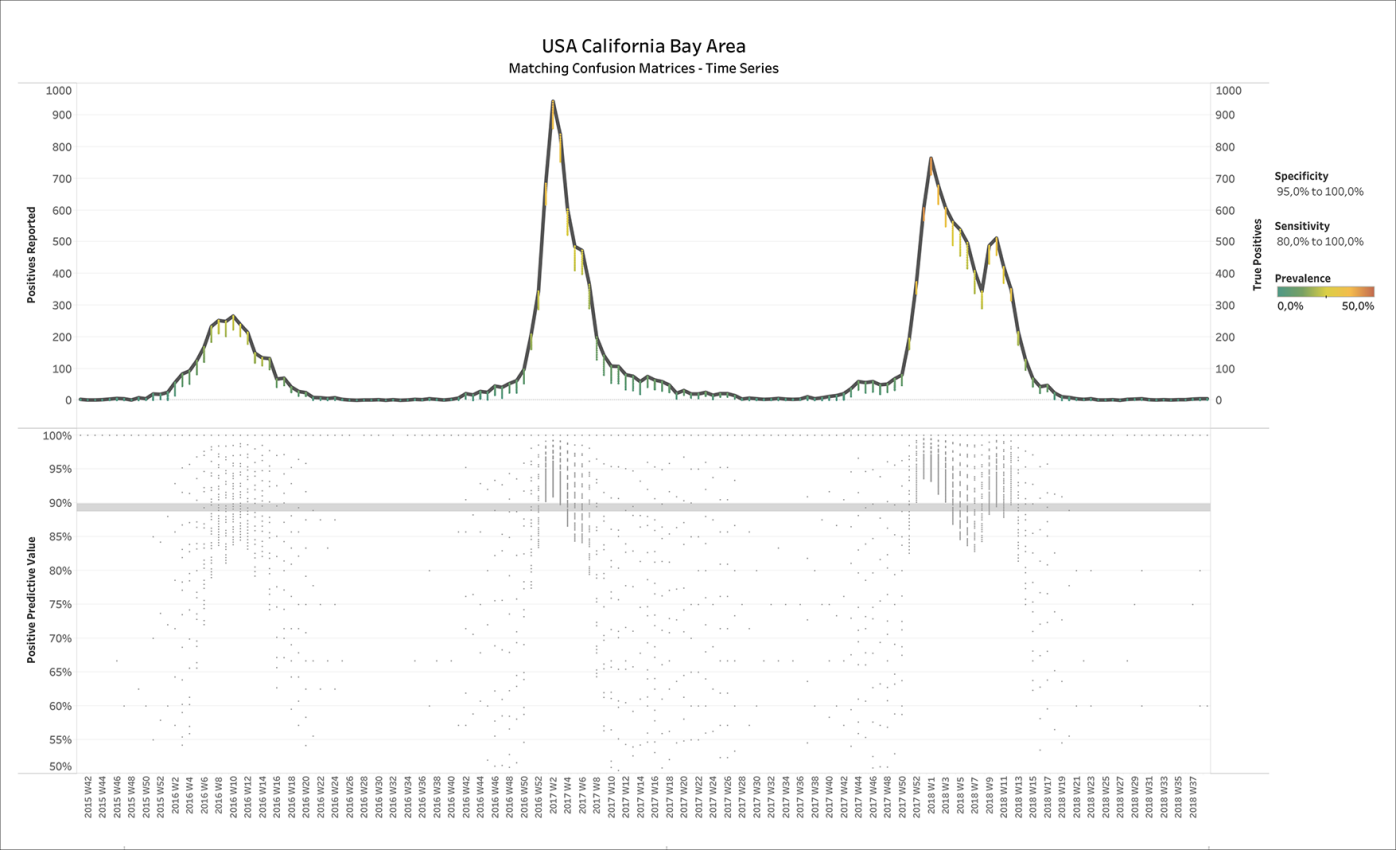
Report ID day vs. positives reported and true positives (upper panel) or positive predictive value (lower panel) for USA-CA-BayArea influenza test data. Upper panel: Color shows details about Prevalence. The view is filtered on specificity and sensitivity. The specificity filter ranges from 95.0% to 100.0%. The sensitivity filter ranges from 80.0% to 100.0%.

Figure 2 shows the results of applying BLT to weekly influenza test data from the Californian Bay Area, USA. The upper panel displays the number of positive tests reported over time, where the estimated number of TPs is overlaid in small dots (CMs) whose color represents the estimated prevalence (see legend on the right of Figure 2). Filters have been applied on specificity (95.0% - 100.0%) and sensitivity (80.0% - 100.0%). One could see that the number of TP tests is close to number of positives reported, except for some deviations during the spring and summer months when prevalence was estimated correctly as low.

The lower panel shows the positive predictive value (PPV), for each CM, defined as $\mathrm{PPV}=\frac{\mathrm{TP}}{\mathrm{TP}+\mathrm{FP}}$ , which confirms a high accuracy of the tests: The median PPV of all CMs over time was almost 90%.

### 3.3 The Netherlands (diagnostic COVID-19 testing)

**Figure 3.**
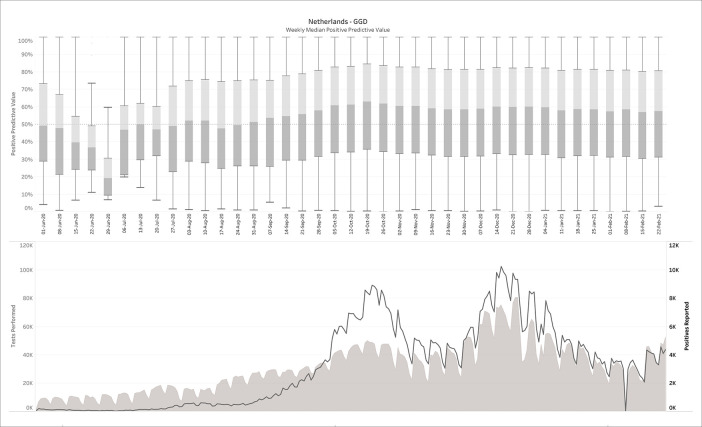
The Netherlands - June 1 2020-Feb 24 2021, weekly median positive predictive value (upper panel), in comparison with tests performed and positive tests (lower panel). No filters on prevalence, specificity or sensitivity were applied here.

269 daily reports were downloaded from the Dutch government Corona dashboard and processed with the SQL-query. This resulted in 809,830 CMs matching the daily reports from June 1 ^st^, 2020 until Feb 24 ^th^, 2021. The upper panel of Figure 3 plots the median PPV, with the corresponding number of performed and positive tests plotted in the lower panel. Note that the left and right y-axes in the lower panel are on different scales.

**Figure 4.**
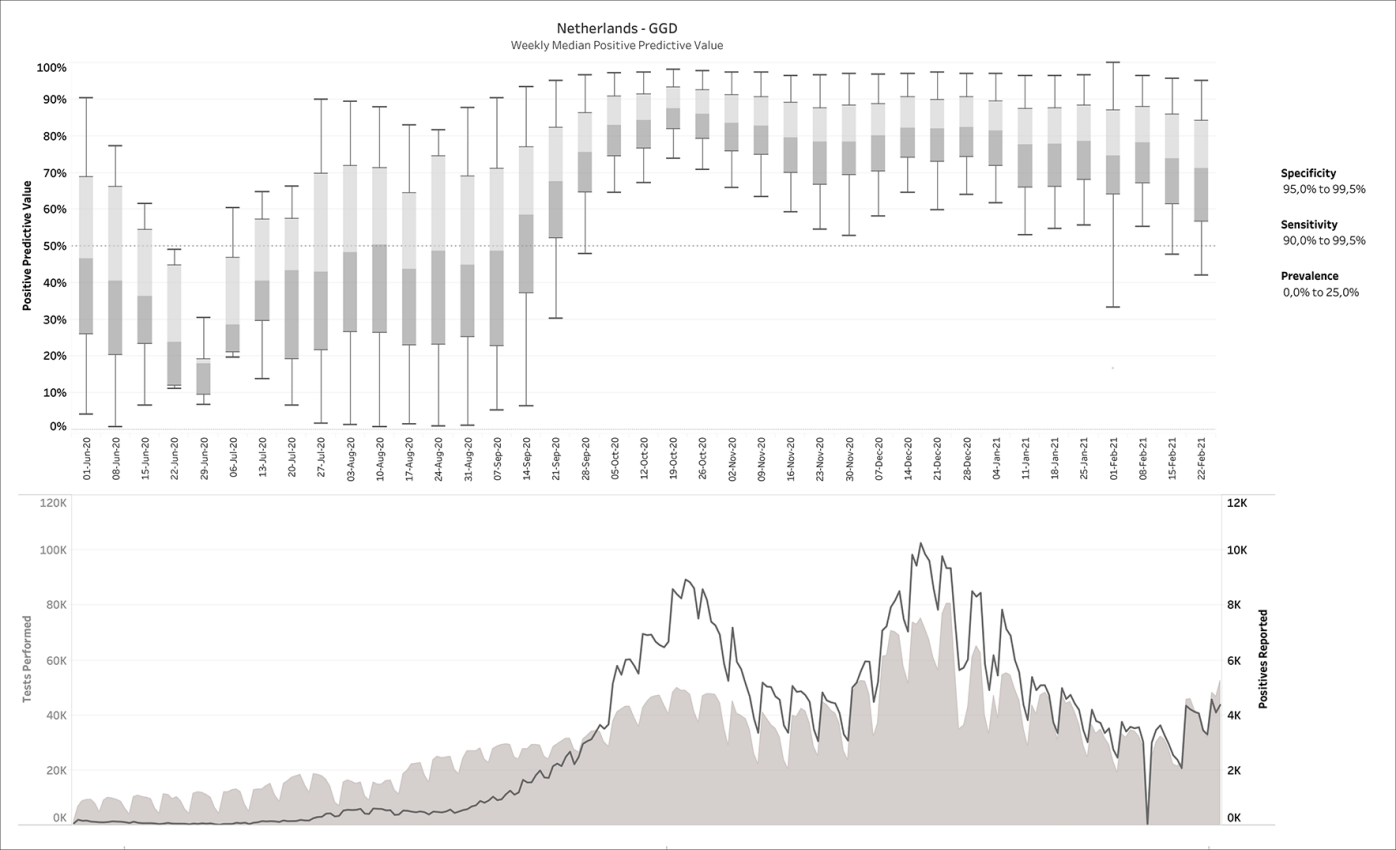
Same as Figure 3, but now with filters applied. The example above shows the 40,200 possible CMs that fit the given report, for 90.0% ≤ sensitivity ≤ 99.9% and 95.0% ≤ specificity ≤ 99.5% and 0 ≤ prevalence ≤ 20%.

It can be observed that in contrast to the influenza example ( Figure 2), the PPVs are now much lower, with a median average around 50%. For this estimation, no filters were applied on sensitivity, specificity or prevalence. When *a posteriori* knowledge is available about the diagnostic tests and/or the circumstances in which they were performed, different scenarios can be applied to the output. This is exemplarily visualized in Figure 4, in which some reasonable filters for a SARS-CoV-2 testing environment have been applied. Notice how the PPV started to increase sharply from a median around 50% before mid-September 2020 to 80-90% during the fall and winter.

**Figure 5.**
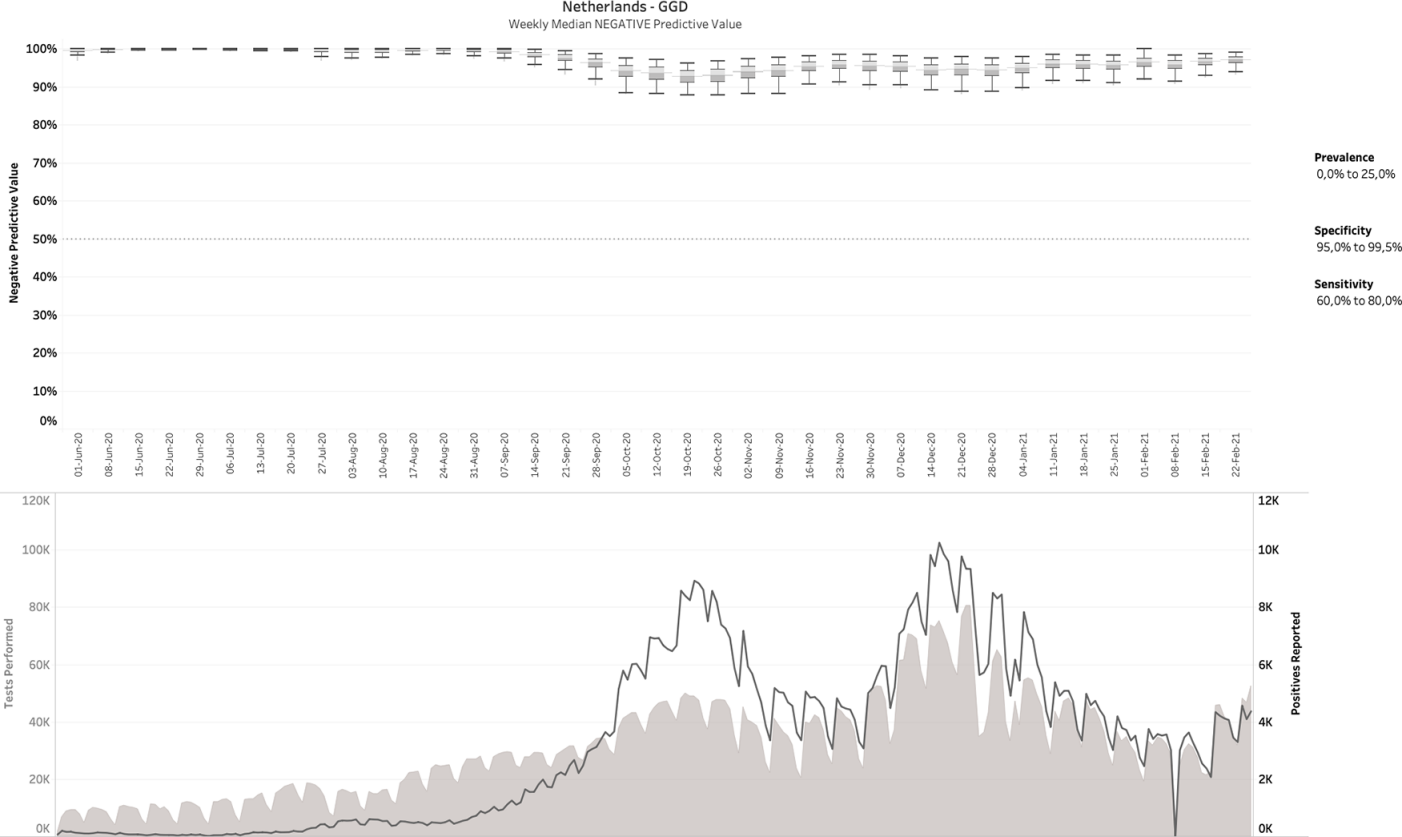
Same as Figure 4, but now plotting NPV and changing the range for sensitivity to 60-80%.

Finally, Figure 5 shows the negative predictive value (NPV) for the Netherlands data with similar filters as in Figure 4, except for choosing a less optimistic sensitivity range of 60-80%, which is consistent with some clinical data. It can be noticed that NPV remains relatively high throughout the entire time range. Median NPV over time does not drop below 90%, even after reducing the range for sensitivity to as low as 60-80%. We also tested the impact of this lower sensitivity range on the PPV, but could not detect any visible impact, consistent with the finding that low-specificity tests cannot distinguish between the hypotheses that a positively tested individual is infected with SARS-CoV-2 or not regardless of sensitivity. ^14^

### 3.4 Germany - Bavaria (diagnostic COVID-19 testing)

**Figure 6.**
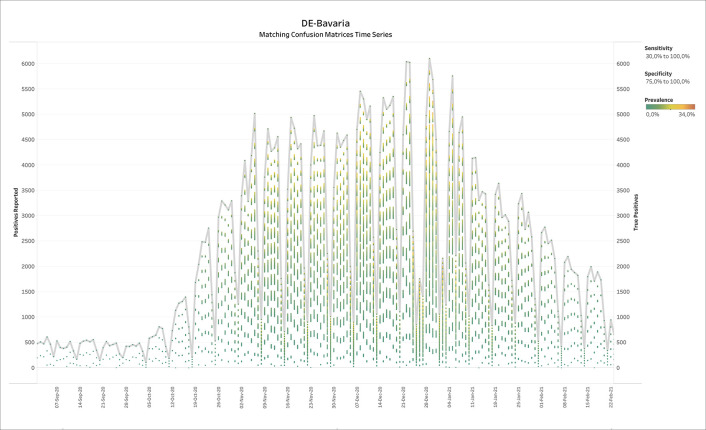
Bavaria, Germany - Weekly reports with positives reported and true positives calculated by BLT. For the true positives, the color shows details about prevalence. The Specificity filter ranges from 75.0% to 100%. The Sensitivity filter ranges from 30.0% to 100.0%. Reports range from week ending 26th February 2020 until week ending 17th February 2021.

Figure 6 shows the output of BLT applied to weekly SARS-CoV-2 testing data from Bavaria in Germany. The thick grey line displays the number of positive tests reported over time, while the colored batches show the solutions of BLT for the TP numbers according to prevalence. Note that in low prevalence scenarios, the TPs do usually not come close to the reported number of positives. At the end of the summer, the prevalence values compatible with the official test reports suggested low prevalence, but also a discrepancy between the number of positive tests and TPs, suggesting a large number of FPs.

## 4. Discussion

The developed Bayesian calculator tool allows the estimation of possible values for the essential variables’ prevalence, sensitivity, and specificity for a specific period of time (e.g., daily or weekly, depending on the input data the user supplies). The solutions provided by BLT are derived from Bayes’s theorem ( Equation 1) under the assumption that $P\left(T\right)=\frac{\#\text{positives}}{\#\text{tests}}$ and $P\left(I|T\right)=\frac{\mathrm{TP}}{\#\text{positives}}$ . In cases of low total and positive test numbers, these assumptions might not hold exactly, but BLT should nevertheless find close solutions to the actual test performance measures. As our applied examples show, the strength of BLT lies in its application to mass testing scenarios such as those conducted during the SARS-CoV-2 crisis.

The BLT calculations are unbiased in the sense that they use all possible and sensible combinations of prevalence, sensitivity, and specificity, and let Bayes’ theorem decide which combinations match the actually observed data. The result for a given matching combination of these three particular parameters is provided in the form of a CM which contains the TP, TN, FP, and FN numbers. In the case where more than one combination is compatible with the given input data, the user may start simulating different scenarios, e.g., by applying prior knowledge regarding the expected prevalence range on a given date. This enables the user to further constrain the combinatorial possibilities of the output variables.

Prevalence is a crucial factor for any inferences based on diagnostic tests, even though it is often not taken into account in practice. This results in the so-called base-rate fallacy. ^19^ Our calculator may result in several possible prevalence values that are compatible with the observed data. In this case, knowledge about the population that has been tested may be used to constrain the possibilities. In 2020, for instance, prevalence-values in the range 12-15% were estimated for German hotspot regions, ^20, 21^ while prevalence was zero in an asymptomatic German mother-and-child population tested in April 2020. ^22^ In an early COVID-19 related publication which compared RT-qPCR to chest computer tomography in 1014 COVID-19 patients from the Tongiji hospital in Wuhan, China, prevalence appeared to be very high: in total 830 patients were described to be confirmed or highly likely to have COVID-19, and of those 580 were diagnosed by chest CT and RT-qPCR and another 250 by CT and clinical decision. These results suggest a prevalence of 81.9% in these patients. A recent preprint publication ^23^ aimed at estimating the sensitivity and specificity of the Chinese RT-qPCR tests by a Bayesian model incorporating information from both chest CT and clinical decision classification. The author obtained sensitivity of 0.707 (95% CI range: 0.668-0.749) and specificity of 0.851 (95% CI range: 0.774-0.941). Applying BLT to these data and assuming that only the cases in which both chest CT and RT-qPCR came out positive (i.e., filtering on 580 TPs), our model reveals a sensitivity of 65.3% and specificity ranging from 83.1%-83.6%, not too different from the estimates of the more complex analysis. ^23^

During the SARS-CoV-2 crisis an unprecedented mass testing not only of symptomatic, but also asymptomatic cases emerged as a strategy. One would expect the prevalence to be substantially higher in the former than in the latter population. As our scenarios 3 and 4 from section 3.1 shows, if there is a mixture of two populations with very different prevalence values, the resulting overall prevalence is a weighted average, provided that the sensitivity and specificity of the tests is similar in both populations.

Our results confirm the recent World Health Organization (WHO) statement “that disease prevalence alters the predictive value of test results; as disease prevalence decreases, the risk of false positive increases”. ^24^ This means that the probability that a person who has a positive result (SARS-CoV-2 detected) is truly infected with SARS-CoV-2 decreases as prevalence decreases, irrespective of the claimed specificity of the test system. ^24^ This statement may be more accurately described as the number of TPs decreasing relative to a constant FP rate so the ‘risk of false positives’ only increases relative to the TP numbers, but the FP frequency is assumed to remain constant across a given number of tests. However, multiple modes of error may be in play. We should not assume FPs are independent of contamination from TP samples. There are higher risks of contamination in rapidly growing laboratories. Contamination of samples in the low disease prevalence seasons (summer) will go unnoticed as they do not produce a qPCR signal. Contamination prone methods may only become evident in the form of elevated and perhaps falsely assumed TPs once the disease prevalence increases in the winter.

In light of the current WHO statement, the rationale for mass testing strategies implemented during periods of low prevalence (e.g., summer) appears questionable. Furthermore, mass testing increases the risk of poor sample handling and laboratory contamination which might partly explain the high FP numbers our calculator predicts. For example, Patrick *et al*. argued that besides intrinsic test performance, amplicon contamination due to high throughput processing of samples within a laboratory would be the best explanation for an increased rate of FP detections made during an outbreak of the human coronavirus HCoV-OC43 in a Canadian facility. ^25^

While much attention has been placed on population frequency of disease and its impact on false positives, it is critical to understand the role of false negatives and the impact these can have on track and trace systems. The nasal swabs are known to vary tremendously in RNaseP Ct values suggesting highly variable sampling or limited RNA stability in the testing reagent chain. ^26^ Woloshin *et al.* demonstrate 27-40% FNs with nasopharyngeal and throat swabs respectively and underscore the importance of understanding pre-test probabilities when interpreting qPCR results. ^27^ These FN numbers are probably not due to the PCR itself, but are related to handling issues and the above discussed problems, as well as the time point within the course of infection that the sample is taken. In a meta-analysis of clinical data, Kucirka et al. found that the probability of a FN test was 100% at day 1 of an infection with SARS-CoV-2 (prior to symptom onset), and then decreased to 38% (95% credible interval 18-65%) at the day of symptom onset down to its minimum of 20% (12-30%) three days after symptom onset, after which it rose again to 66% (54-77%) three weeks after the infection. ^28^ Hence, according to these numbers, even in infected individuals sensitivities below 30% are possible, a range that we excluded in our analysis consistent with Klement and Bandyopadhyay. ^14^ This points to additional problems when testing asymptomatic individuals, because in case that they are truly infected, a high number of FNs is going to result.

With the script presented here, we can think of many variations when it comes to the range of sensitivity and specificity, their step-sizes (granularity) and the ‘where’ clause as well as the strictness of matching TP+FP against the reported positives. For example, one could also increment prevalence on a log-scale to account for the fact that prevalence in many settings of diseases is very low. ^14^

We are aware that choices made in these areas have a significant impact on the number of matching CMs. An impact/sensitivity analysis was not performed, although we suspect that such analysis might reveal additional insights. However, we think that the amount of matching CMs per result that the above query produces delivers sufficient material to make useful observations.

Future research with different data-repositories, for instance ECDC/TESSy-data would be very beneficial to identify a solid balance between precision (step-size in the permutations), number of matching CMs, and overall query performance.

## 5. Conclusions

We have developed an easy-to-use Bayesian calculator (Bayes Lines Tool, BLT) to estimate prevalence, sensitivity, and specificity, and therefore TP, TN, FP, and FN numbers, from official test outcome numbers. With typical reports - especially as produced for SARS-CoV-2 tests - revealing just the number of positives and number of tests performed, the BLT SQL implementation generates CMs that fit within the boundaries of a typical simplified report, based on permutations of sensitivity and specificity. Its implementation is thereby not limited to SQL but can be applied on any platform of choice.

The ability to assess posterior probability independent of the circumstances in which diagnostic tests are performed, reveals a wide spectrum of opportunities for new applications both for the scientific community as well as for health professionals and policy makers around the globe. This is especially relevant for the mass testing taking place within the containment strategies of worldwide governments against the SARS-CoV-2. The BLT SQL query for the first time allows one to display a real estimation of the SARS-CoV-2 situation against the background of testing volume and quality and thus will provide a valuable tool for decision makers to monitor the test strategy and the effect of interventional procedures.

This tool will not only allow official institutions to survey the test situation and obtain a better basis for planning their interventions, but also allows for individuals who got tested to use the confusion matrices as an aid for interpreting their test results in view of the population they were tested in.

## Data availability

### Underlying data

All data underlying the results is linked in section 2.3 of the article. The hypothetical example is given in Table 2. No additional source data is required.

## Software availability

Zenodo:

Bayes Lines Tool (BLT) - A SQL-script for analyzing diagnostic test results with an application to SARS-CoV-2-testing, http://doi.org/10.5281/zenodo.4594210. ^29^

Code is available under the terms of the Creative Commons Attribution 4.0 International license (CC-BY 4.0).

The SQL-code and an example implementation in Excel and a Tableau work-book file can be downloaded at https://bayeslines.org/.
