# Supplementary material for: Bayes Lines Tool (BLT): a SQL-script for analyzing diagnostic test results with an application to SARS-CoV-2-testing
Source: F1000Res. 2022 Feb 16;10:369. Originally published 2021 May 10. [Version 3] doi: 10.12688/f1000research.51061.3 (PMC8891718; doi:10.12688/f1000research.51061.3)
Supplement: Peer review history

# Peer review: Reviewer response for version 3

**DOI:** 10.5256/f1000research.121230.r123945

**Published:** 2022-03-02

**Authors:** Mariska M G Leeflang

**Affiliations:** 1 Department of Epidemiology and Data Science, Amsterdam UMC, University of Amsterdam, Amsterdam, The Netherlands

I have nothing to add - my concerns were already addressed in a previous version.

Are the conclusions about the tool and its performance adequately supported by the findings presented in the article?

Yes

Is the rationale for developing the new software tool clearly explained?

Partly

Is the description of the software tool technically sound?

Yes

Are sufficient details of the code, methods and analysis (if applicable) provided to allow replication of the software development and its use by others?

Partly

Is sufficient information provided to allow interpretation of the expected output datasets and any results generated using the tool?

Partly

Reviewer Expertise:

Clinical epidemiology and evaluation of medical tests

I confirm that I have read this submission and believe that I have an appropriate level of expertise to confirm that it is of an acceptable scientific standard.

# Peer review: Reviewer response for version 2

**DOI:** 10.5256/f1000research.57358.r91450

**Published:** 2021-10-04

**Authors:** Mariska M G Leeflang

**Affiliations:** 1 Department of Epidemiology and Data Science, Amsterdam UMC, University of Amsterdam, Amsterdam, The Netherlands

The authors developed a what they call 'Bayesian calculator' to estimate false and true positives and negatives from reported test results. They state that this will be a useful tool for health professionals and policy makers, and even for individuals to interpret their own test results. 

 As I am not familiar with SQL-code and would have no idea how to implement this calculator on my own computer, I thought that the authors partly provided "sufficient details of the code, methods (…) to allow replication of the software development". So I have not checked for mistakes in the code, or whether the calculator actually works. It would be really helpful if someone could do that.

 Technically speaking, the formulas and the explanations all seem to be in order. I have little to comment on that. However, there are a few semantics-issues that may be resolved, and the rationale of a separate calculator is not entirely clear to me.

**Some more specific comments:**

1. Using the phrase 'Bayesian calculator', implies to me that Bayesian statistics have been used, and that the factors the authors mention in their abstract and introduction (sample handling, underlying test protocols etc.) have been taken into account to go from a prior belief about sensitivity/specificity/prevalence to a posterior (after accounting for other factors) belief. However, when reading the manuscript, it turns out that the BLT is nothing more than a huge number of permutations given a starting value of positive and negative results. It provides a range of possible true values, without providing an indication of how realistic all these possibilities are. Therefore, I think the authors are overselling a relatively simple calculator and that the new thing of this BLT is actually only the way the data are presented. I can do these calculations in Excel as well, but that would give me a headache to provide the right figures and graphs. So maybe the whole article should tone down the novelty a bit.
2. In my previous comment I mentioned the lack of information about how realistic some predictions may be. Would it be possible to add this information?
3. If I were a policy maker and I would get Figure 1 out of this program, how should I interpret the results and how should I implement the information in my policy making? I am missing the link with practice. Could the authors maybe provide some instructions about what the results mean and what their implications for practice maybe?
4. I find abbreviations and acronyms confusing, although that may be a personal thing. For example, CM for confusion matrix is only one word less in the word count every time CM is being used. But using the full term is much more informative and easier to read.
5. I am not sure whether the examples given (20% prevalence in the general population) are realistic. I think the true prevalence of SARS-CoV-2 infections has been much lower at any given point in time.
6. The authors stated that: "Our results confirm the recent World Health Organization (WHO) statement “that disease prevalence alters the predictive value of test results; as disease prevalence decreases, the risk of false positive increases”." However, this is an inherent given for predictive values. It is in their calculation. So this statement follows from logic, while the way it was written here, it implies that the authors have proven this WHO statement to be correct. And it implies that this is a recent finding. Both are not true. So please rephrase it. Maybe using language explaining that your findings follow the premise that prevalence alters the predictive value.
7. I think it is a missed opportunity that the authors have not performed a sensitivity or impact analysis. Now it is just showing us how the SQL code works, but it does not provide us further insights into where results may go wrong, or when they become more or less reliable.

Are the conclusions about the tool and its performance adequately supported by the findings presented in the article?

Yes

Is the rationale for developing the new software tool clearly explained?

Partly

Is the description of the software tool technically sound?

Yes

Are sufficient details of the code, methods and analysis (if applicable) provided to allow replication of the software development and its use by others?

Partly

Is sufficient information provided to allow interpretation of the expected output datasets and any results generated using the tool?

Partly

Reviewer Expertise:

Clinical epidemiology and evaluation of medical tests

I confirm that I have read this submission and believe that I have an appropriate level of expertise to confirm that it is of an acceptable scientific standard, however I have significant reservations, as outlined above.

# Peer review: Reviewer response for version 2

**DOI:** 10.5256/f1000research.57358.r87900

**Published:** 2021-08-27

**Authors:** Phillip M. Bentley

**Affiliations:** 1 European Spallation Source ESS, Lund, Sweden

The article has been amended according to review 1 and should be indexed in its current form.

Are the conclusions about the tool and its performance adequately supported by the findings presented in the article?

Yes

Is the rationale for developing the new software tool clearly explained?

Yes

Is the description of the software tool technically sound?

Yes

Are sufficient details of the code, methods and analysis (if applicable) provided to allow replication of the software development and its use by others?

Yes

Is sufficient information provided to allow interpretation of the expected output datasets and any results generated using the tool?

Yes

Reviewer Expertise:

Physics, data analysis, simulations, computer modelling, game theory, strategy.

I confirm that I have read this submission and believe that I have an appropriate level of expertise to confirm that it is of an acceptable scientific standard.

# Peer review: Reviewer response for version 1

**DOI:** 10.5256/f1000research.54169.r84995

**Published:** 2021-05-18

**Authors:** Phillip M. Bentley

**Affiliations:** 1 European Spallation Source ESS, Lund, Sweden

The authors have done a great job and this article should be indexed after addressing the issues below:

- Equation 6 has a typing mistake.
- The authors state on page that "These FN numbers are probably not due to the PCR itself, for which sensitivity is almost 100% ( https://www.finddx.org/covid-19-old/ sarscov2-eval-molecular/), but a matter of handling issues and the above-discussed problems." This is incorrect. The false negative rate of the PCR test is documented as a function of time (see ref 1), ^1^ and is mostly related to low early virus shedding initially. The middle phase, when the test has the lowest false negative rate, is as the authors describe, but then the lack of virus particles as the patient recovers becomes the major factor. Laboratory-based validation of PCR testing, as cited by the authors, differs from the clinical FN as seen in the present study due to potential flaws in the entire sample collection, handling, and processing chain. It is important to consider the whole chain in evaluating error rates. This is perhaps the most important aspect of test errors, since FP results in some inconvenience and/or worry whilst FN provides a dangerous false sense of security.
- The analysis of Bavaria (figure 5) is roughly consistent with the numbers reported by Kucirka, but those of the Netherlands (figure 4) suggest a sensitivity range that is highly optimistic. The specificity on the other hand could be very good depending on the regional expertise.
- The authors need to address the difference between their data and what would be expected from Kucirka *et al.*
- The negative predictive value is very important for governments/regions to release healthy people with confidence. The
  authors should present this number as they have done for the positive predictive value.

Are the conclusions about the tool and its performance adequately supported by the findings presented in the article?

Yes

Is the rationale for developing the new software tool clearly explained?

Yes

Is the description of the software tool technically sound?

Yes

Are sufficient details of the code, methods and analysis (if applicable) provided to allow replication of the software development and its use by others?

Yes

Is sufficient information provided to allow interpretation of the expected output datasets and any results generated using the tool?

Yes

Reviewer Expertise:

Physics, data analysis, simulations, computer modelling, game theory, strategy.

I confirm that I have read this submission and believe that I have an appropriate level of expertise to confirm that it is of an acceptable scientific standard, however I have significant reservations, as outlined above.

## References

[1] : Variation in False-Negative Rate of Reverse Transcriptase Polymerase Chain Reaction–Based SARS-CoV-2 Tests by Time Since Exposure. *Annals of Internal Medicine* .2020;173(4) : 10.7326/M20-1495 262-267 10.7326/M20-1495 32422057PMC7240870

# Response

**Published:** 2021-06-15

**Authors:** Rainer Klement

**Affiliations:** Leopoldina Clinic, Germany

We thank Dr. Bentley for his time to evaluate our article and constructive comments that we think have led to further improvements. We hope that the revised version can now be approved.

 Below, we reply to each of Dr. Bentley’s points. The revisions to the text have been marked with track changes in MS Word.

  

- Equation 6 has a typing mistake.

**Answer: Thank you for pointing it out, we advised the F1000 team to correct it.**


**  **

- The authors state on page that "These FN numbers are probably not due to the PCR itself, for which sensitivity is almost 100% ( https://www.finddx.org/covid-19-old/ sarscov2-eval-molecular/), but a matter of handling issues and the above-discussed problems." This is incorrect. The false negative rate of the PCR test is documented as a function of time (see ref 1), ^1^ and is mostly related to low early virus shedding initially. The middle phase, when the test has the lowest false negative rate, is as the authors describe, but then the lack of virus particles as the patient recovers becomes the major factor. Laboratory-based validation of PCR testing, as cited by the authors, differs from the clinical FN as seen in the present study due to potential flaws in the entire sample collection, handling, and processing chain. It is important to consider the whole chain in evaluating error rates. This is perhaps the most important aspect of test errors, since FP results in some inconvenience and/or worry whilst FN provides a dangerous false sense of security.

**Answer:  Thank you for pointing out the time factor relative to the time of infection and the study by Kucirka et al. We have changed the text into the following: “** **These FN numbers are probably not due to the PCR itself, but are related to handling issues and the above discussed problems, as well as the time point within the course of infection that the sample is taken. In a meta-analysis of clinical data, Kucirka et al. found that the probability of a FN test was 100% at day 1 of an infection with SARS-CoV-2 (prior to symptom onset), and then decreased to 38% (95% credible interval 18-65%) at the day of symptom onset down to its minimum of 20% (12-30%) three days after symptom onset, after which it rose again to 66% (54-77%) three weeks after the infection. Hence, according to these numbers, even in infected individuals sensitivities below 30% are possible, a range that we excluded in our analysis consistent with Klement and Bandyopadhyay. This points to additional problems when testing asymptomatic individuals, because in case that they are truly infected, a high number of FNs is going to result.”**

**We try to avoid judgements such as yours (“since FP results in some inconvenience and/or worry whilst FN provides a dangerous false sense of security“), which may invoke subjective arguments regarding “inconveniences". We acknowledge that this is the general perception about FN versus FP and will elaborate further on this, with your last comment / point. We appreciate that you point this out and will add an additional figure to prevent any suggestion of bias in the article.** 

  

- The analysis of Bavaria (figure 5) is roughly consistent with the numbers reported by Kucirka, but those of the Netherlands (figure 4) suggest a sensitivity range that is highly optimistic. The specificity on the other hand could be very good depending on the regional expertise.

**Answer:** **When reducing sensitivity towards a less optimistic range of 60-80%, we see no visible impact on PPV in figure 4, probably because sensitivity has no significant influence on PPV. This is now described in words in Section 3.3.**

  

- The authors need to address the difference between their data and what would be expected from Kucirka *et al.*

**Answer: A brief discussion has been added, in line with the answer to your point above: “** **Hence, according to these numbers, even in infected individuals sensitivities below 30% are possible, a range that we excluded in our analysis consistent with Klement and Bandyopadhyay. This points to additional problems when testing asymptomatic individuals, because even if they are infected, the resulting FN numbers may provide a false sense of security.”**


** **

- The negative predictive value is very important for governments/regions to release healthy people with confidence. The
  authors should present this number as they have done for the positive predictive value.

**Answer: Thank you for pointing this out. We understand and agree that NPV should be presented as prominently as PPV and will do so by adding a new figure for the Netherlands, taking in consideration your remarks about the sensitivity range being set too optimistically. Notice how NPV remains relatively high throughout the entire time range. Median NPV over time does not drop below 90%, even after we reduce the range for sensitivity to as low as 60-80%.**
